# Myopraktikk (NO): A Narrative Review and Conceptual Hypothesis on Intrafasciomembranal Fluid Pressure, Biotensegrity, and Immediate Remote Myofascial Responses

**DOI:** 10.7759/cureus.93711

**Published:** 2025-10-02

**Authors:** Stig Runar Hopen, Tor Arne Mjøen

**Affiliations:** 1 Physical Medicine and Rehabilitation, The International Myopraktikk Organization, Beitstad, NOR; 2 Research, The International Myopraktikk Organization, Beitstad, NOR

**Keywords:** biotensegrity, intrafasciomembranal fluid pressure, muscle knots, muscle tension, myalgia, myofascial pain syndrome, myopraktikk (no), psychosocial pain, tension equalization, trigger points

## Abstract

This article introduces Myopraktikk (NO), a Norwegian-developed manual treatment method, where we clinically observe by palpation and visual inspection immediate local and remote responses that form the basis for a new hypothesis uniting intrafasciomembranal fluid pressure (IFMFP), Myopraktikk treatment chains, and the principle of biotensegrity in a single framework.

Here, IFMFP refers to fluid pressure within fascial compartments, treatment chains describe functional pathways of force and tension that appear to respond collectively to local input, and biotensegrity denotes mechanical balance in an interconnected network.

Muscle pain and myalgias are among the most common causes of pain and functional impairment, yet current models remain fragmented and leave major gaps. Why does nonspecific pain and stiffness arise without structural findings? Why do muscles feel “tight” at rest, and why do some tensions resolve quickly while others persist? What are muscle knots, trigger points (TrP), and fascial restrictions in regional pain conditions, and how might they connect to fibromyalgia (FM), overtraining syndrome (OTS), myalgic encephalomyelitis/chronic fatigue syndrome (ME/CFS), post-traumatic stress disorder (PTSD), or even everyday complaints such as morning stiffness and weather-related pain?

The IFMFP model postulates fluid accumulation and pressure rise within fascial spaces surrounding muscle fibers, bundles, and whole muscles, driven by overuse and impaired drainage, and conceptually comparable to intramuscular compartment pressure. This pressure alone might shorten and thicken tissue, and induce venous stasis, hypoxia, pH reduction, hyaluronan (HA) modification, and altered membrane permeability. Unlike traditional theories that see HA changes as causal, the IFMFP model proposes the reverse: pressure drives HA alterations and densification, enabling chronic dysfunction.

A key clinical observation is that, by palpation and visual inspection, knots, TrP, tensions, and fascial cords are often felt to diminish during treatment, both locally and remotely, with patients and practitioners reporting perceived immediate changes in asymmetry, tonus, and stiffness. These findings suggest such structures are not static lesions but dynamic fluid pockets of increased IFMFP, dissolving as pressure is released.

Based on clinical observations, the Myopraktikk treatment chains reveal patterns of movement, force, and tension, where local interventions may influence the entire chain through systemic equalization, in line with biotensegrity.

By integrating biomechanical, physiological, and psychosocial factors, IFMFP offers a unifying mechanism that may link diverse myalgias within an expanded framework of myofascial pain syndrome. Myopraktikk therefore challenges established assumptions both as a treatment method and as a conceptual framework, where immediate, systemic, and reversible changes in myofascial tension, amplified by the physical expression of stress and thought, provide a fresh understanding of myalgias.

## Introduction and background

Muscle pain, myalgias, and tension-related complaints are common causes of functional impairment and reduced quality of life in the population [1]. Despite this, there is still considerable uncertainty regarding the underlying mechanisms, which may lead to uncertain classification and varying treatment strategies. Many patients experience long-lasting or recurrent pain in muscles and connective tissue, without any clear findings in imaging examinations or laboratory tests [2-5]. Such patient courses call for a more holistic understanding of the body’s regulatory mechanisms, particularly in cases where the symptom profile lacks conventional findings yet still involves a high degree of subjective pain, complaints, and loss of function [2]. The role of fascia and fascial manipulation has received attention in recent research [6,7].

Despite extensive research, it remains unclear why many patients experience nonspecific pain and stiffness without structural findings, why muscles can feel “tense” at rest, and why some tensions are short-lived, while others become chronic. Phenomena such as muscle knots, trigger points (TrP), and fascial restrictions lack a unified explanation, both in common conditions, such as low back pain, neck pain, and tennis elbow, and in more complex disorders, such as fibromyalgia (FM), overtraining syndrome (OTS), myalgic encephalomyelitis/chronic fatigue syndrome (ME/CFS), and post-traumatic stress disorder (PTSD). Nor is it clarified whether the same mechanisms can explain everyday complaints such as morning stiffness and weather-related pain, or whether overlapping symptom profiles point to a common etiology.

Myopraktikk (NO) is a Norwegian-developed manual treatment approach and conceptual model that has emerged through more than a decade of clinical experience and close patient observation (not to be confused with myopractic, which is a different tradition with a different origin). The development, conceptual framework, and clinical mapping of Norwegian Myopraktikk have been carried out by myopraktors (NO), Anne Grete Johnsen and the main author of this article [8].

The treatment is primarily intended for adults with musculoskeletal pain and impaired function, including athletes. It is not recommended for children, pregnant women, or patients with cancer or other serious medical conditions, as safety in these groups has not been established. Contraindications include bleeding disorders, anticoagulant therapy, recent surgery, and unexplained cysts or masses. In cases of uncertainty, treatment is only undertaken in collaboration with the patient’s physician, and if clear contraindications are present, patients are referred to their physician for follow-up.

The treatment is directed toward pain and impaired function in the musculoskeletal system and is based on clinical observations such as increased tonus and palpable structures in the myofascial tissue, often described as “knots,” “nodules,” “threads,” or “cords,” as well as general hypertonic muscle tensions, all of which may present as visible swellings on the skin surface [1,8]. Although such observations are not captured by traditional diagnostic tools, they appear clearly to both patient and practitioner during clinical palpation [2,8]. Both patients and myopraktors report that such nodules and hypertonic tensions often disappear instantly during treatment. These observations are based on palpation, the disappearance of visible swellings on the skin surface, and the patient’s report of pain relief when the previously painful area is palpated again after treatment [8], thereby challenging traditional explanatory models based on structural damage or tissue alterations. These clinical observations led to a theory of fluid pressure dynamics [1,8,9].

Furthermore, repeated observations of immediate, systemic, and often remote treatment responses made it necessary to seek an explanation. Tensions disappeared not only locally, but also in other parts of the body, raising the question of how such effects could propagate. In this context, the concepts of biotensegrity, fascintegrity, and myofascial chains appear as central [10]. Thus, a holistic explanatory model emerged, in which treatment of one area could, in practice, create immediate and systemic changes by releasing tensions and muscle knots, and reducing pressure in one region, with simultaneously clear corresponding effects in other parts of the body [8,9].

The purpose of this article is to compile and present the framework behind Myopraktikk, intrafasciomembranal fluid pressure (IFMFP), and biotensegrity, illuminated through these responses. By integrating clinical observations with theoretical models, a new perspective is advanced on muscle-related pain and functional disorders, their possible common etiology, and their placement within the umbrella concept of myofascial pain syndrome (MPS). This forms a foundation for further research, interdisciplinary dialogue, and a potential paradigm shift in the understanding and treatment of such complaints.

## Review

Myopraktikk: From clinical experience to systemic understanding

This narrative review is based on more than a decade of clinical experience with Myopraktikk (since 2012-2013) and a non-systematic literature search (mainly PubMed, with additional sources from colleagues), with emphasis on articles on intrafasciomembranal fluid pressure (IFMFP) [1,9] and the Norwegian textbook Myopraktikk - Behandlingskjeder: Teori og behandling (Myopraktikk Treatment Chains - Theory and Treatment) [8].

Myopraktikk (NO) is a deep-acting manual treatment approach based on systematized clinical observations and experiences. It combines manual treatment, physiological explanatory models, and the body’s capacity for self-regulation. The method is described in more detail in the Norwegian textbook on Myopraktikk [8]. In short, once contraindications are excluded, treatment is performed with the patient in a prone or supine position, depending on the area being treated. Rapid crosswise traction is applied to specific points along defined treatment chains, often in the scapular musculature. Fingers are used in smaller and more localized areas, while the elbow is applied in larger muscle groups and when deeper pressure is required.

Typically, reciprocating movement is performed per point with the elbow, while finger applications are often repeated several times. The force ranges from moderate to firm but never more than necessary, depending more on technique than on strength. It is always adjusted in agreement with the patient, who is encouraged to provide feedback and retains full control to stop the treatment at any time. Each stroke lasts only a few seconds, with sufficient pauses between applications. Transient bruising may occur. Although rapid traction could theoretically carry some risk, it is considered low when performed briefly and within the patient’s tolerance. In more than a decade of clinical experience, no persistent neurovascular injuries have been observed. Myopraktikk is generally intended as a single-session treatment, with ample time devoted to assessment and patient education to provide insight and tools for independence. The manual part of the treatment typically lasts a total of 15-30 minutes, depending on the patient’s need for pauses.

Manipulation is applied across the muscle fiber direction, a crosswise traction, targeting specific points within anatomically anchored and functional Myopraktikk treatment chains, defined by the body’s natural load patterns [8]. According to the textbook, many of these points are located in the musculature of the scapula, but each treatment chain extends across larger regions of the body that appear to respond and function as one integrated treatment unit [8].

Patients often seek treatment for pain, stiffness, or loss of function in muscles and joints without knowing the cause. Clinically, palpable and often painful structures are observed, which the patient recognizes and which correspond with their complaints [1]. Some are aware of such tensions, while others relate only to the pain or loss of function. The tensions are often accompanied by visible swellings or asymmetries that reinforce the clinical picture [8].

In order to understand what these palpable structures actually were, and how such tensions arise, fluid dynamics and an overlooked mechanical fluid pressure were identified as IFMFP, a key factor in the understanding [1]. Even before our own understanding of IFMFP was established, clinical observations pointed to a deeper connection: patients and practitioners often reported immediate relief, also in distant areas of the body. This has formed the basis for ongoing efforts to obtain objective before/after measurements. Over time, it became evident that these remote effects were not coincidental but followed recurring patterns related to which structures were treated. This provided the basis for a gradual systematization of the observations, the origin of the Myopraktikk treatment chains [8].

Myopraktikk therefore represents not only a treatment technique, but a new explanatory model for pain and tension. It is based on the idea that many complaints arise from universal mechanisms that overload the body’s fluid-regulating systems and manifest as distinct tensions in the tissue.

IFMFP: A new understanding of myalgias

The IFMFP model represents an important theoretical advance in the understanding of myalgias and myofascial pain conditions [1,9]. The model forms part of a growing understanding of the role of fluids in pain [11,12]. It brings together and explains phenomena that have previously been perceived as isolated symptoms and provides a holistic explanation for conditions where traditional diagnostics often fall short [2,11]. It is based on the body’s fascial architecture, where the solid fascia functions as a continuous membrane that both surrounds and infiltrates the muscle tissue.

This fascia forms small and large fluid spaces at different anatomical levels, from muscle fibers (endomysium) to fiber bundles (perimysium), entire muscles (epimysium), and muscle compartments (osteofascial septa) [1]. In more recent times, this has increasingly been understood as functional spaces with their own mechanical and fluid-related properties [1,8,9,11-13]. The fascia that encloses all these spaces thus forms a hierarchy of semipermeable fascial membranes (fasciomembranes), enabling a local accumulation of fluid within such membranes [1]. Regardless of whether the fluid pressure is found in muscle fibers, fascicles, an entire muscle, or a compartment, it is referred to as IFMFP, namely, because the fasciomembranes allow for differentiation at all anatomical levels [1].

In Myopraktikk, increased IFMFP is understood as either a primary cause or an important contributing factor to the clinical picture in a number of myalgias and muscular complaints [1,8,9]. The IFMFP model clarifies how an imbalance between arterial inflow and venous drainage arises, particularly in areas with sustained muscle activation. Prolonged, repetitive, or static overload, as in sedentary work, emotional stress, overuse, or overtraining, can maintain arterial inflow even during more or less conscious muscle contractions, while venous and lymphatic drainage is mechanically impeded. Even small, sustained contractions may be sufficient to disturb the fluid balance in the tissue [1,9].

Compromised Venous and Lymphatic Drainage

During physical activity, muscle compartments can increase in volume by up to 20% as a result of increased blood flow, a response that under normal conditions is reversed at rest [1]. With increased volume due to overuse, whether sustained or repetitive, without sufficient drainage between contractions, compression of capillaries, venules, and lymphatic capillaries occurs, which can hinder normal fluid outflow. The increased pressure impairs the overall drainage of interstitial fluid and waste products. The arterial inflow, which has higher pressure, continues to force fluid into the tissue, leading to a further increase in pressure within the confined fascial space [1]. The mechanism of increased pressure is well documented in chronic exertional compartment syndrome (CECS) [1,14,15], and the same physiological pattern is also found in TrP, OTS, and delayed onset muscle soreness (DOMS) [1].

Doppler ultrasound studies have demonstrated increased outflow resistance near trigger points, indicating a mechanical obstruction of venous drainage [1,16]. Such obstruction may likely worsen fluid stagnation and circulatory dysfunction, and may contribute to the development and maintenance of pressure-related myalgias [1]. A crucial point regarding myalgias is that the obstruction is no longer due to active muscle contraction, but that increased pressure can independently compress venous and lymphatic structures. While contraction normally gives a transient increase in volume that reverses, overuse creates a persistent and inherent pressure. Thus, a passive drainage obstruction arises. Palpable nodules, cords, and muscle tension arise from this perspective only when IFMFP exceeds the tissue’s drainage capacity, resulting in a passive tension without electromyography (EMG) activity. Such tensions are likely mechanically significant even at rest. This suggests that the problem does not necessarily lie in the tissue itself, but in the pressure that develops within it. Anatomical variations in fascial structure and fluid volume mean that increased pressure has different significance from person to person. Because IFMFP can occur to varying degrees and extent, and at all levels of muscle tissue, even small pressure changes can produce clear physiological and symptomatic effects [1]. The absence of structural findings in traditional imaging diagnostics, therefore, does not mean that IFMFP is not real: fluid distribution, microcirculation, and interstitial load are difficult to visualize, especially without comparable before-and-after images. In addition, there are methodological challenges related to direct measurement of pressure in muscle tissue: although pressure in some cases can be measured with a cannula, it varies between different areas within the muscle [17], and the muscles contain a large number of small and large fluid spaces, which makes reliable measurements challenging [1]. The challenges with diagnostics do not, however, diminish the relevance of IFMFP; rather, they make it all the more evident that new models are needed to understand the phenomenon.

Mechanical Stimulation of Pain Receptors

Myofascial tissue is richly innervated, and many free nerve endings (nociceptors) terminate in the interstitial space close to the muscle fibers. These nerve endings respond to both chemical and mechanical stimuli, and their pressure sensitivity means that even small changes in fluid volume and pressure can directly affect them and trigger pain [1]. IFMFP may thus explain why patients with muscular pain often experience tenderness and pain even in the absence of tissue damage or inflammation. Pressure on nociceptors, therefore, appears as a central mechanism underlying the pain experience in several types of myalgias [1,9].

Reduced Microcirculation and Hypoxia (Oxygen Deficiency)

Increased IFMFP leads to local oxygen deficiency in the tissue [1]. This is seen in conditions such as CECS, where ischemic pain, loss of function, and reduced oxygenation of the muscle cells have been documented [1]. Fluid stagnation likely increases resistance in the tissue, thereby reducing the supply of new, oxygen-rich arterial fluid. When the outflow of CO₂-rich and waste-laden fluid is blocked, an imbalance may arise in which new oxygen and nutrients have reduced access, while spent fluid remains trapped. This may further contribute to the hypoxic state and reduced metabolic function, muscle weakness, and fatigue [1]. Hypoxia thus appears as a natural and consistent consequence of elevated IFMFP and constitutes an important component in the further development, laying the foundation for biochemical changes in the tissue.

Decrease in pH: Local Acidosis

Under hypoxia, the muscle cells shift to anaerobic metabolism [18]. This leads to increased production of lactate and pyruvate, two acids that lower the pH in the tissue, cause acidosis, and form the basis for ischemic pain. Such mechanisms could, in the future, be further supported by clinical proxies, for example, near-infrared spectroscopy (NIRS), to assess local oxygenation, although methodological challenges remain. Such biochemical changes have been documented in fibromyalgia (FM), where elevated levels of both interstitial lactate and pyruvate have been measured [1,19]. Acidosis also directly affects muscle function and disrupts the contractile processes in the muscle cells, reducing force generation and contributing to increased fatigue and muscle weakness [20]. This may provide an important contribution to the experience of “heavy,” “sore,” or “dead” muscles in various myalgias.

Changes in Hyaluronan (HA) and Reduced Permeability

As a consequence of such hypoxia and decrease in pH, biochemical changes arise in the extracellular matrix (ECM) of the tissue, particularly related to hyaluronan (HA), a polysaccharide that functions as a central component of the body’s fascia, where it lubricates and hydrates the tissue [21]. Permeability is the ability of a membrane to allow selected molecules, ions, and fluids to pass through it [22]. As semipermeable structures, fasciomembranes play a crucial role in the body’s interstitial fluid dynamics, enabling regulated fluid transport that is essential for tissue homeostasis and cellular function [11]. When the local environment becomes acidic, HA shifts from being viscous and lubricating to becoming sticky and adhesive. This limits both gliding and fluid flow, a condition referred to as densification of fascia [1,9,23]. Since the fasciomembranes are an integral part of the fascial network, it follows that they too are subjected to the same densification, which reduces the membranes’ permeability, reinforces the pressure, and contributes to increased IFMFP. Although the term “reduced permeability” is rarely used in the literature, several studies point to underlying mechanisms that collectively correspond to such a decline in permeability through the fasciomembranes [1,9,21,24,25]. In contrast to traditional theories where changes in HA are attributed a primary role in creating pressure, swelling, and compression [25], the IFMFP model points to a reversed causal sequence: muscle overuse first leads to pressure and fluid stagnation, where reduced circulation causes hypoxia, which lowers pH, alters HA, and reduces permeability. This triggers a self-reinforcing chain of changes that increase pressure, impair tissue mobility, and amplify dysfunction, potentially making the condition chronic. Thus, pressure and drainage failure appear to be the initiating factors behind HA-related tissue changes, not the other way around. Changes in HA and permeability, therefore, emerge as yet another crucial factor in the development and maintenance of pressure-related myalgias. Impaired fluid flow and reduced membrane function emphasize that IFMFP must primarily be understood as a physiological and mechanical process, rather than an inflammatory one.

Mechanical Pressure and Reduced Permeability

Increased fluid volume with a corresponding rise in IFMFP will inevitably lead to increased mechanical tension in the fasciomembrane. Unlike the biochemical HA changes described above, this mechanism is mechanical: pressure physically compresses and reorganizes the tissue and HA molecules. This tension generates compressive forces, alters structure and elasticity, and mechanically reduces the membrane’s permeability. Fasciomembranes consist of a three-dimensional, braided structure of collagen fibers surrounded by a ground substance (ECM). This includes HA, proteoglycans, and glycoproteins, which are central components of connective tissue. In the deep fascia, there is also a distinct layer of HA between the fascia and the underlying muscle [21,26].

As pressure increases, the collagen fibers in the membrane are stretched and reorganized into a more unidirectional orientation [27]. This reduces porosity, that is, the number and size of the openings through which fluid can pass, and thereby also reduces the permeability of the membrane [28]. At the same time, the ground substance is compressed, causing the HA molecules to become more tightly packed, both within the fasciomembrane itself and likely also in the HA layer inside the membrane. This increases viscosity and further slows fluid flow through the fasciomembrane [21,23]. At the cellular level, the connective tissue cells (fibroblasts) register this mechanical stress through mechanosensitive ion channels such as Piezo1 [29]. This triggers signals that cause the cells to remodel and reinforce the tissue, a natural adaptive mechanism for modeling and stabilization. When this process persists, the result is a self-reinforcing “locking effect,” where both collagen architecture and HA viscosity limit fluid flow. Thus, the pressure is maintained, and the process results in a form of densification that is anchored mechanically and biochemically, and at the cellular level.

Increased Pressure Expressed as Tonus and Stiffness

The relationship between increased pressure and muscle tonus appears to be close and dynamic, supported by research showing that pressure directly affects both tonus and stiffness [30,31]. Increased tonus and stiffness characterize many myalgias and functional muscle disorders, but the underlying mechanisms are often unclear in the literature. The IFMFP model is based on the premise that elevated IFMFP mechanically affects the properties of the tissue and provides a convincing explanation for both increased tonus and stiffness in the musculature [1,8,9]. A similar mechanism is also seen in inflammatory conditions that cause swelling and increased fluid volume in the tissue, which inevitably raises the local pressure [30]. Similar to a balloon being filled with more water, the increased pressure in such a closed space will lead to higher passive tension and thereby increased muscle tonus and stiffness [9].

This supports that IFMFP is a central regulator of tonus and stiffness, and helps explain why muscles may be experienced as “tense,” “heavy,” or “shortened,” thereby contributing to compensatory movement.

Densification and Long-Term Stiffness

Thickening of the fascia can be regarded as the tissue’s natural adaptation to increased load from muscle use over time. With repeated exposure to such stress stimuli, fibroblasts will gradually adapt the morphology of the tissue [32]. Densification involves increased density in the tissue, leading to mechanical stiffness and reduced flexibility in the affected area [21,23]. Muscle overuse thus generates increased mechanical pressure and structural changes in HA, derived from IFMFP, thereby providing a valid explanation for the development of densification [9]. Such changes provide a basis for viewing long-term stiffness, increased tonus, and densification as physiological expressions of persistently elevated IFMFP. The proposal that pressure can independently maintain fluid accumulation even without muscle activity reinforces the understanding of these mechanisms as central in the development and maintenance of long-term myofascial stiffness [1,9]. Fibrosis and densification share several features, such as increased ECM density, reduced gliding, and alterations in HA. Although fibrosis is often considered more permanent, the similarities suggest that they may share mechanisms or represent different stages in a common process triggered by prolonged mechanical or metabolic stress [21,23].

Veterinary research shows similar changes in animals, such as “wooden breast” in broiler chickens. Here, rapid growth leads to fibrosis, reduced blood supply, and thickened tissue [33]. Other animal studies show that prolonged negative stress can alter both pH and produce a tougher structure in muscle tissue. Psychological stress factors appear to have a more detrimental impact on tissue quality than purely physical loads [34]. This supports that various types of overload, stress, and mental strain can contribute to IFMFP-driven tissue changes. The same principle is widely recognized in animal research and everyday experience: stressed animals develop tougher, stringy meat. Clinically, humans can present a parallel, where muscle and connective tissue under persistent stress can be palpated as stiff, tough, or thickened. Prolonged IFMFP, with continuous internal mechanical pressure, may thus trigger such processes. Over time, this can lead to densification, reduced elasticity, and long-term stiffness, not only as a result of altered fluid viscosity and HA structure, but as an expression of a slow, adaptive remodeling of connective tissue, possibly progressing all the way to fibrosis.

IFMFP and Systemic Propagation

The fiber architecture of the muscle changes dynamically during voluntary contraction, and ultrasound studies have demonstrated both shortening and increased muscle depth [9]. This is caused by pressure buildup as the shape of the muscle changes and is illustrated by an increased cross-sectional dimension combined with reduced length, an effect reminiscent of Poisson’s ratio. Increased IFMFP can produce similar shape changes even in the absence of active contraction, where muscle tissue shortens and thickens as a result of fluid accumulation [1,9]. A muscle fiber or muscle enclosed by a fasciomembrane can be understood through the analogy of a balloon within a spindle-shaped net, which illustrates the mechanism simply: when the balloon is inflated, the pressure and diameter increase while the length shortens, precisely because the net resists the internal expansion. In the same way, increased IFMFP can produce mechanical shortening and transverse expansion in muscle tissue [9]. Forces from this shape change propagate through the myofascia and can exert a mechanical push on adjacent structures. Shortening in the IFMFP-affected area simultaneously creates stretch and increased mechanical traction in neighboring tissue along the longitudinal axis, which thereby loses some of its own depth [9]. This may lead to local compression of blood and lymphatic vessels as well as nociceptors, with a risk of ischemia, metabolic impairment, and mechanical pain within this tissue as well [1,8,9]. Over time, this persistent load may also lead to structural adaptations within this tissue. The fascia may both thicken and alter its gliding properties, which can create altered lines of force through the body, affecting posture and promoting compensatory patterns of movement [1,8,9].

The transmission of both compressive and tensile forces in myofascial structures opens a new perspective on referred pain. A typical example is TrP, where a taut band contains a hyperirritable spot. In light of IFMFP, this can be understood as a local area with elevated pressure and tissue shortening (the trigger point itself), which creates traction along the fiber direction (the taut band) and thereby activates nociceptors along an adjacent region of myofascia [9]. This also provides a valid explanation for the phenomenon of “knots” and “threads” upon palpation. Mechanically, IFMFP can be compared to a muscle contraction, but in contrast to a short-term voluntary activation, the elevated pressure exerts a continuous force that gradually alters both the form and function of the tissue.

This fluid-induced tension can thus propagate through myofascial chains, where both tensile force and pressure affect both local and surrounding tissue [1,9]. Increased pressure and mechanical stress along the body’s natural lines of tension may thus reduce the permeability of fasciomembranes, primarily for interstitial fluid, and exacerbate stagnation across regions and over time, although this remains challenging to document in vivo. Nevertheless, it supports the hypothesis underlying one of the key principles of the IFMFP model: that elevated IFMFP does not act only locally, but can spread systemically through myofascial connections and biotensegrity, contributing to persistent, widespread systemic tension states [9].

Biotensegrity

Traditional biomechanics views the body almost like a machine built from rigid structures, bones and joints, moving as levers and hinges driven by muscles, where movement is primarily explained by isolated forces and linear transmission. The concept of biotensegrity, derived from “biological tension and integrity,” introduces a contrasting and more holistic understanding, in which the body’s structures, including bones, connective tissue, and muscles, function as a dynamic, self-stabilizing network. Here, mechanical tension from connective tissue and muscles is balanced against passive compression from bones, continuously throughout the body. This provides both flexibility and stability and explains how local loading can have global effects, thereby also offering a foundation for manual treatment [35].

Several models provide a theoretical representation of active and passive forces in the human body through a fascial continuum. The models of biotensegrity, fascintegrity, and myofascial chains are all based on valid concepts [9,10].

Biotensegrity entails that the transmission of mechanical tension (active or passive) leads to constant adaptation of body structure and posture, without damaging or deforming the body’s form and function [10,35]. Simple tensegrity models of rods and elastic bands provide an illustration of how applied forces propagate and distribute throughout the structure. In the same way, biotensegrity functions as the body’s shock absorber by relieving local loads through holistic tension distribution. This concept is applicable throughout the entire body, and the principles apply both to contractile muscles and down to individual cells [35].

A myofascial chain is a continuous tissue line of muscles and connective tissue (fascia) that has been mapped through dissection and follows an anatomical direction through the body [36]. We further know that the myofascial chains are layered and lie on top of each other. These chains are also more or less connected to each other by connective tissue, fascia, or interstitium with their fiber directions, primo vessels, interstitial spaces, nerves, vessels, and tissue fluids [8]. Several of these chains also converge to joints and bones as common anchoring points.

Fascintegrity also includes tensions arising from nervous tissue, vascular tissue, and the movement of body fluids (liquid fascia), such as blood, lymph, and interstitial and intracellular fluid [10,37].

These models do not exclude each other, but rather show the complexity and complement each other. They explain how tension in one contractile area can affect others, both nearby and distant. Cadaver and in vivo studies support such mechanical transmissions [38]. This suggests that biotensegrity influences the myofascial chains and vice versa. Together, they constitute a fascial continuum that enables the transmission of chemical, electrical, and mechanical signals and forces.

Myopraktikk treatment chains

**Figure 1:**
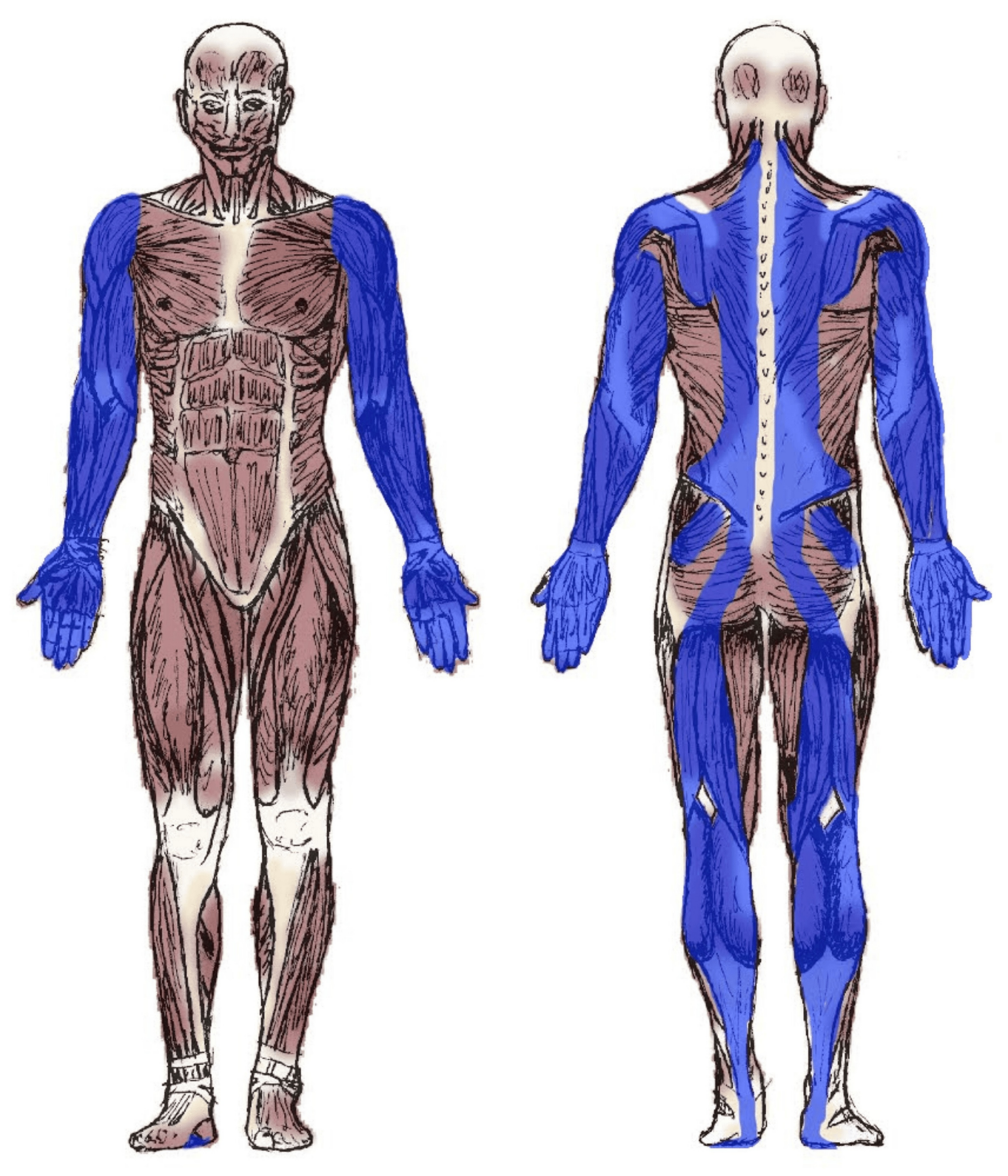
Typical pathway of the infraspinatus muscle treatment chain, one of the largest guiding Myopraktikk treatment chains Adapted from Myopraktikk - Behandlingskjeder: Teori og behandling [8] Author’s own publication, used with permission

Through the principles of biotensegrity and myofascial chains, it becomes possible to understand the functional Myopraktikk treatment chains, a concept presented in the Norwegian textbook in connection with the body’s inherent architecture for movement and stability, where communication, tension, and resistance propagate along natural structural patterns in the tissue [8,9]. It explains how tension, pressure, and load can be distributed throughout the body, and why local influence can create responses in distant areas through Myopraktikk manipulation. It is natural to think that the body continuously attempts to distribute tension as evenly as possible, as part of a dynamic self-stabilizing process [35], a process that enables stability, balance, adaptation, and function in the entire musculoskeletal system, and ultimately is what keeps us upright. Moreover, muscles, fascia, and connective tissue interact to distribute such force, pressure, and tension through the tissue’s directions and structural connections [9,10,35,39]. This can be compared to a tent, where tension in one guyline affects the entire structure. When one region of the body is exposed to load or overactivity, this may result in displacements, overcompensations, or secondary tensions in other areas (Figure 1).

Thomas Myers has previously described myofascial chains in his concept Anatomy Trains [9,10], but the Myopraktikk treatment chains differ from this by being based on clinical response rather than on anatomical connection alone [8]. Since muscles, fascia, nerves, and other structures often share connective tissue and cooperate to create movement and stability, several myofascial chains can be part of an integrated system of tension and load. In light of this, it becomes easier to understand how muscle tension from increased IFMFP in one myofascial chain creates restrictions and dysfunction along the chain, while at the same time, IFMFP may also compress or pull on chains located in the layers above or below. This can in turn produce a domino effect in the adjacent chains [8]. The phenomenon can be understood as a form of referred pain or load, where the cause may lie distant from the symptom [9].

The systematization of repeated observations of which areas trigger pain or functional change in other parts of the body and which points during treatment produce effects in distant regions makes the treatment chains appear as a functional and empirically founded map of the body’s patterns of tension and relief, rather than as merely dissected anatomical chains. This repeatedly observed interaction forms both the practical and theoretical basis for the treatment chains in Myopraktikk. It has been crucial for the development of a holistic physiological model that explains the body’s mechanical response patterns in light of internal pressure changes and provides a framework for how tension states may arise, propagate, and be influenced through treatment [1,8,9]. This was, as mentioned, eventually understood through the concept of biotensegrity [10,35,39].

A total of seven guiding treatment chains of varying length and extent have been defined, each named after the muscle that is primarily manipulated [8]. These are understood as distinct pathways for the distribution and regulation of tension throughout the body, and are as follows: infraspinatus treatment chain, trapezius treatment chain, latissimus dorsi treatment chain, deltoideus treatment chain, teres major treatment chain, teres minor treatment chain, and iliopsoas treatment chain.

The chains may overlap but together encompass the entire body. Each treatment chain has both a right and a left side. Clinical observations indicate that a balanced bilateral response is rare, whereas treatment most often produces a distinct unilateral effect. However, manipulations may still produce some direct and secondary effects on the opposite side [8]. Collectively, all the treatment chains function as the body’s overall tensional system, but are experienced as independent “domains of action” during treatment. The treatment chains form the practical map of areas that respond as a unit and are therefore used as the basis for assessment and treatment [8].

**Figure 2:**
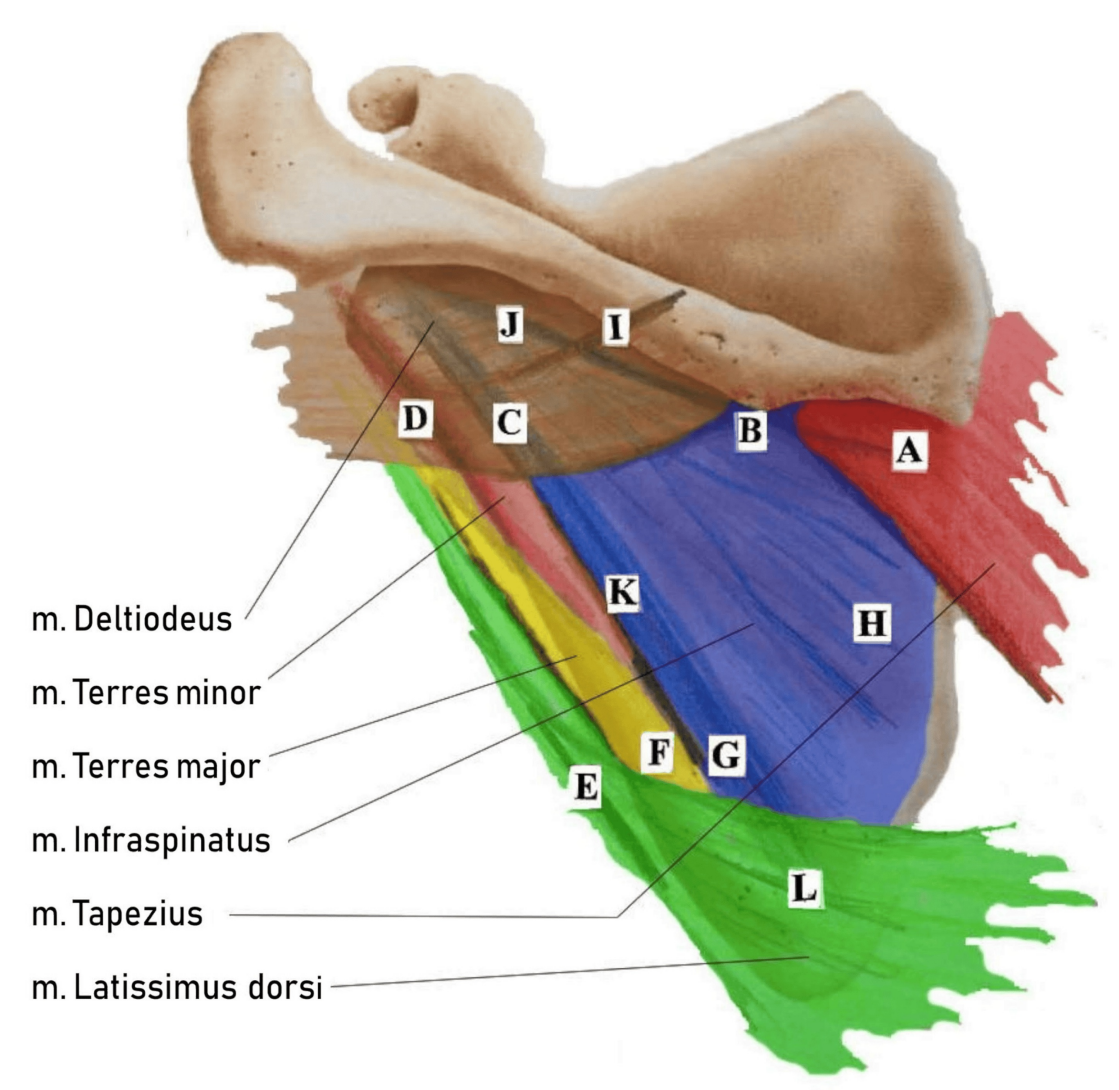
Scapula with associated muscles and marked treatment points Color coding indicates the muscles’ affiliation to different treatment chains. Adapted from Myopraktikk - Behandlingskjeder: Teori og Behandling [8] Author’s own publication, used with permission

The scapula is central in Myopraktikk, as most treatment chains converge here and include treatment points on muscles overlying the scapula, rendering the area particularly suitable for influencing several chains simultaneously [8]. The scapula is also anatomically well-suited for powerful manual treatment. Although the infraspinatus, teres minor and major, deltoid, trapezius, and latissimus dorsi are anatomically distinct, together, they form a densely integrated muscle mass over the scapula below the spine of the scapula. This common anchoring provides increased stability and predictability during manipulation. At the same time, the scapula protects the underlying structures, which ensures a high degree of safety during treatment, although it may be perceived as painful due to local nerve innervation [8]. Functional centrality and anatomical robustness make the scapula a strategic and safe foundation for Myopraktikk intervention. Clinical practice indicates that treatment points on the scapular muscles can trigger systemic palpable changes, including in the arms, neck, back, and as far down as the calves (Figure 2).

The location and character of muscle tension are important for both symptom expression and treatment response. In some cases, deep or compensatory tensions may be hidden, which may explain why the effect of treatment can vary. The proposal that targeted treatment of one point can provide immediate relief and functional change at a considerable distance from the intervention itself nevertheless reflects the body’s ability to respond systemically to local treatment and emphasizes that neither tension, load, nor the perception of pain is necessarily localized to the same site. Since both the body’s architecture and tension patterns vary individually, the defined treatment chains must still be regarded as guiding rather than absolute. By understanding the body as a dynamic system with functional Myopraktikk treatment chains, this provides a framework for thinking and treating holistically, a tool for acting systemically, yet with precision.

Factors leading to IFMFP

The IFMFP model describes a process in which several factors may create functional imbalance in the tissue [1,9]. Overuse, through static load, repetitive use, or psychosocially induced tension, is central. When muscle use exceeds capacity, reduced fluid flow may contribute to myalgias [1,9]. Overtraining, pain-induced muscle contraction, static work, and stress can all lead to sustained muscle activation. What unites them is continuous contraction and a physiological imbalance, regardless of whether the load is due to physical activity or not [1], often also without the patient being aware of the increased tension.

An important reflection is where the boundary lies between normal physiological adaptation, such as increased muscle volume and tissue adaptation through training, and the transition to dysfunction. The IFMFP model suggests that what initially represents a healthy response to load may shift into a pathological state when fluid accumulation and pressure become persistent, and when low pH and densification interfere with the body’s ability to maintain normal regulation and function.

Psychological Stress and Physiological Changes

It is widely accepted that events and negative stress are stored in the body, yet this notion often lacks explanatory substance regarding the process; what, in fact, is actually stored in the body? If we look more closely at negative stress, it is natural to understand this as the body’s reaction to burdens that become overwhelming or prolonged. Stress may arise from many sources: work or school, family or social challenges, financial problems, or physical factors such as temperature, sleep deprivation, illness, or persistent pain. In addition, trauma, major life events, or self-imposed demands and expectations, combined with a lack of coping and recognition, may contribute to negative stress.

Individuals exposed to significant negative stress will mobilize into a prolonged “fight, flight, freeze” response, in which the body unconsciously mobilizes and keeps the musculature activated in order to react to danger. This involves elevated blood pressure, increased heart rate, and a redistribution of blood from the digestive system to the brain and muscles [40,41], something that in itself may contribute to increased IFMFP.

However, it is important to keep open the possibility that other mechanisms may contribute. Thoughts and emotions have physiological substance, through hormones, neurotransmitters, and nerve signals, and may thereby worsen or maintain states of tension, a driving factor in pathological development.

Research on rats also shows that psychological stress can affect muscle tissue in a similar way as physical load, and metabolic changes, including higher levels of lactic acid in the muscles, have been observed [42]. This suggests that psychological stress can lower pH and alter HA, leading to densification and reduced permeability in fasciomembranes, a mechanism that may also be relevant in humans.

Although not all factors are fully understood, both physical and psychosocial mechanisms are likely involved in persistent low-grade muscle activation that increases IFMFP. This may explain why some patients do not respond to traditional rest or unloading, as the elevated pressure is maintained by unconscious muscle activation even during apparent rest. A high level of negative stress may also explain why some patients improve during treatment but relapse when returning to their daily stress patterns. Ultimately, this provides a valid explanation for why pain and discomfort often appear “without cause” and why many experience that things are “stored in the body.” IFMFP thus offers a tangible and plausible physiological explanatory model, even for this.

Clinical spectrum and pathophysiological understanding

Clinical observations over many years provide compelling indications that pressure-related mechanisms appear to explain a range of diagnoses and conditions with shared characteristics, pointing to IFMFP as a possible underlying and unifying factor, or a contributing factor, in such conditions [1,9]. The IFMFP model helps explain why different conditions share symptom patterns. How various complaints manifest depends on the degree of pressure, the anatomical level, the affected area, any spread to neighboring tissue, the duration, and possible secondary conditions. Within this understanding, tension, generally increased tonus, and palpable muscle knots are not seen as isolated problems, but as symptoms, the body’s own physiological way of signaling that something is wrong, whether through overload, stress, or functional imbalance. This perspective shifts the focus from symptom to cause, opening the way for more targeted and meaningful treatment.

Despite increasing insight into pathophysiology and treatment, many unanswered questions remain in the literature regarding the etiology and systemic nature of different forms of myalgia. Conditions such as TrP, CECS, OTS, and delayed onset muscle soreness (DOMS) are often classified as separate entities, but previous articles have suggested that they may share overlapping and underlying mechanisms through IFMFP, including a likely connection to MPS [1,9].

One of the most clinically relevant implications of the IFMFP model is its broad applicability. When the body is understood within the framework of Myopraktikk, IFMFP, and biotensegrity, it opens for an explanatory model that encompasses a wide range of pain conditions, including many that previously have been difficult to understand and diagnose. Several of these conditions are associated with reported clinical phenomena and practice-based observations that, in light of the IFMFP model, can be regarded as different manifestations of the same mechanism, terms such as muscle tension, muscle knots, TrP, or the designation “myose” used in Scandinavia [1,43]. These terms are often used interchangeably and may describe anything from entire muscles and muscle groups to very small and localized areas. Other commonly used descriptions include hypertonic, edematous, or “packed” muscles [1]. Many of these conditions, such as TrP, CECS, OTS, and DOMS, are characterized by muscular tension, tenderness, reduced function, and absence of structural findings and have therefore often been classified as “functional,” psychosomatic, or idiopathic. In many cases, it is the pain, whether diffuse and generalized, or localized and distinct, that forms the basis for the patient’s diagnosis or complaint, without either the patient or the healthcare system being able to find a satisfactory explanation for the underlying mechanisms. Within the framework of IFMFP, however, such conditions can instead be understood as physiological expressions of pressure-dynamic disturbances in the body, based on shared, more or less distinct, cardinal symptoms such as swelling, pain, and loss of tissue function [1,9].

IFMFP should be understood as a dynamic and gradable phenomenon, where conditions may also be deep, diffuse, or so general that they are easily overlooked, especially when they do not present as distinct palpable structures. Such conditions manifest more as a general sense of pain, tension, or discomfort across larger areas of tissue.

Another perspective that should be emphasized in relation to clinical presentation is that muscular tensions may produce symptoms closely resembling pain from structural injuries; MRI and X-ray can reveal degeneration and damage in joints and tendons, but also in individuals who report no pain. Studies have shown that between 25% and 80% of certain joint changes occur in people without symptoms [44-47]. This means that what is seen on imaging is not always the cause of the pain and that the experienced pain may instead be related to muscular tensions and functional imbalances in the tissue that stabilize and control the joint. Even when imaging shows degeneration or injury, the reduction of such surrounding tensions may then result in the structural changes no longer giving rise to clinically significant symptoms.

IFMFP and inflammation also produce similar symptoms, and reflecting on this distinction can be useful. In inflammation, fluid accumulation results from an immune response with inflammatory mediators. In IFMFP, the pressure primarily arises from mechanical fluid stagnation without necessarily involving inflammation. In practice, a gradual overlap may be conceivable, where pressure could trigger degrees of secondary inflammation, and inflammation could in turn increase pressure. The definition, therefore, becomes crucial in clarifying where IFMFP ends and inflammation begins.

Taken together, this provides the basis for a significant expansion of the conditions in which IFMFP may be considered a relevant explanatory model.

Myofascial Pain Syndrome as an Expanded Umbrella Term

MPS is classified as a form of rheumatism and represents the most common cause of musculoskeletal pain [48]. MPS is an umbrella term for regional and systemic pain, often with characteristic TrPs and muscle tension. The diagnosis is made clinically based on typical symptoms, pain, local tenderness, palpable tension, and referred pain, and frequently supported by the absence of structural findings on imaging [48-51]. By applying the IFMFP perspective to conditions that are currently classified under other diagnostic labels but share similar symptom patterns, an expanded understanding of MPS becomes possible. This perspective suggests that these conditions may represent variants of the same underlying physiological mechanism, thereby sharing a common pathophysiological basis across diagnoses. From the standpoint of the IFMFP model, it then becomes natural to use MPS as an umbrella term for a broader spectrum of myalgias and pain syndromes. It is claimed that MPS affects approximately 30%-50% of patients with musculoskeletal disorders [48], but with such an expanded understanding, the actual prevalence may potentially be considerably higher.

Regional Muscular Pain and Strain-Related Conditions

A number of common muscle pain and overload conditions are characterized by localized pain, tenderness, and loss of function, often without detectable findings on imaging. When both TrPs and more general muscle tensions are understood as expressions of increased IFMFP, with corresponding effects on surrounding tissue, a more comprehensive clinical picture emerges.

Research shows a high prevalence of active and latent TrPs in back pain [52], and an association between a tight diaphragm and nonspecific low back pain [53]. In shoulder disorders, treatment of TrPs has demonstrated symptom reduction [54]. In tension-type headache, increased pain sensitivity in the neck muscles is common, and treatment of TrPs in the neck-shoulder region has shown improved neck posture and a marked effect on headache frequency [55]. In elbow and forearm conditions such as repetitive strain injury (RSI), tennis elbow, and carpal tunnel syndrome, tension and increased IFMFP in the forearm and scapula may explain persistent symptoms [56-60]. In plantar fasciitis, a strong correlation has been found between tight gastrocnemius and pain severity [61]. Temporomandibular disorders (TMDs) typically show increased tension and TrPs in the jaw, neck, and shoulder musculature [62]. Joint pain often presents with increased tonus and tension in surrounding musculature, which may indicate pressure effects and mechanical strain, contributing to pain development even without joint damage [54,63]. Chest pain misinterpreted as cardiac problems is, in many cases (up to around 50%), caused by TrPs and muscle tension in the chest muscles [64] and can be explained by the IFMFP model.

Systemic Pain Disorders and Complex Syndromes

These conditions are characterized by widespread pain, fatigue, and loss of function, often here as well without structural findings. Within the IFMFP model, this can be understood as systemic disturbances in fluid and pressure dynamics across larger myofascial regions, leading to nerve irritation and ischemia, along with corresponding broader effects on surrounding tissue.

In FM, pain is likely due to hypoxia and persistent muscle contraction, not inflammation [65]. Low-grade, sustained muscle activation and poor drainage may explain persistent high tonus, stiffness, and pain. As direct support, metabolic dysfunction with elevated lactate and pyruvate has been documented [19], and intramuscular pressure has been measured at nearly three times higher in FM patients compared to healthy controls [66], findings that support key aspects of the IFMFP model. Post-traumatic stress disorder (PTSD) results in sustained sympathetic activation and muscle tension. One study shows a higher prevalence of trigger points in war veterans with PTSD [67], with a clear connection to pain and myofascial tensions.

Myalgic encephalomyelitis (ME) and chronic fatigue syndrome (CFS) were previously regarded as separate conditions but are now increasingly referred to collectively as ME/CFS due to overlapping symptoms [68]. Furthermore, CFS is also associated with FM, and a clear common feature here is tender trigger points over muscles or tendon attachments [69], which may explain both pain and reduced function. In clinical practice, many patients with both ME/CFS and FM also report cognitive problems and brain fog [68]. A possible contributor to these symptoms is reduced glymphatic drainage, the brain’s system for clearing waste products, which drains through the jugular veins in the neck and depends on both deep sleep and low muscle tonus [70,71]. With sustained tension in the neck and throat, both venous and subsequent glymphatic drainage may be impaired, which can contribute to increased intracranial pressure and neuro-symptoms [72,73]. This may imply disturbed waste clearance from the brain, potentially explaining symptoms such as brain fog, concentration difficulties, and fatigue in both patients with FM and ME/CFS, a secondary consequence of IFMFP.

As previously proposed in the IFMFP model, both OTS and DOMS indicate patterns of increased muscle tension, reduced drainage, and fluid stagnation, supporting a pressure-based explanation [1].

Irritable bowel syndrome (IBS) shows a strong association between stress and abdominal pain [74]. Muscle tension in the pelvic and abdominal regions and fluid disturbances in the connective tissue around the intestines may, in line with the IFMFP model, represent a common underlying mechanism that, depending on individual vulnerability and local response, can give rise to different symptom expressions, including both IBS and nonspecific abdominal pain. In endometriosis, tissue outside the uterus creates local inflammation, pressure, and pain. Tension in surrounding muscles and fluid disturbances may also exacerbate the symptoms [75].

Polyneuropathy and small fiber neuropathy [76] can partly be explained and understood as increased pressure and fluid accumulation around peripheral nerves, which may impair nutrient supply and cause pain, tingling, and numbness. Similar mechanisms may be relevant in early rheumatic conditions without visible inflammation.

Idiopathic complex regional pain syndrome (CRPS) shows clear signs of inflammatory microcirculatory changes and edema [77], making IFMFP disturbances a plausible part of the mechanism.

Weather-related pain, reported in conditions such as FM and headache, may be due to already elevated tissue pressure being aggravated when atmospheric pressure drops. The IFMFP model explains this as a pressure difference influencing symptoms during low-pressure conditions [9]. When the temperature rises, the three-dimensional structure of HA also gradually breaks down [78], making the tissue more pliable and potentially explaining why some experience less stiffness and pain in warmer weather.

Pressure-Related Conditions

Pressure-related disorders also involve fluid accumulation, reduced drainage, and increased tissue pressure. In CECS, physical activity leads to increased pressure in muscle compartments, resulting in pain and numbness [1,8,9]. The IFMFP model can also be extended to an even broader perspective, recognizing similar mechanisms in other fasciomembranal compartments as well.

Idiopathic intracranial hypertension (IIH) is caused by increased pressure in the brain without a known cause. Reduced drainage and venous compression are mentioned as triggering factors [72,79], and the IFMFP model suggests that tension in the neck and cervical region may contribute to such pressure increases through mechanical compression of the jugular vein, which is linked to reduced glymphatic drainage. Chronic tension in the neck and cervical region may therefore contribute to both increased intracranial pressure and impaired waste clearance from the brain, potentially explaining symptoms such as brain fog and concentration difficulties. Narrowing of the internal jugular vein is also associated with several central nervous system disorders, such as Ménière’s disease (a chronic disorder of the inner ear with episodes of vertigo, hearing loss, tinnitus, and a sensation of pressure), and has also been linked to Alzheimer’s disease [73].

Glaucoma is a leading cause of irreversible blindness and, at the outer limits of the IFMFP framework, can be regarded as a condition with similar mechanisms. Findings include reduced drainage of aqueous humor through the trabecular meshwork of the eye and significant tissue stiffness [80]. Densification-like mechanisms may be a contributing factor here, and mechanical influence from surrounding tissue may affect the drainage, opening the door for an IFMFP-based interpretation of the disorder.

Edema and tissue tension in inflammation illustrate how fluid accumulation produces pain and pressure sensitivity. Many patients receive anti-inflammatory treatment, often without effect [81], which may be due to the fact that they actually have pressure-based disorders that are non-inflammatory and fall outside current diagnoses. The IFMFP model thus points to a potentially overlooked problem: that many patients with pressure-related pain, stiffness, and swelling are misdiagnosed and mistreated because fluid disturbances in fascial compartments are not part of conventional medical understanding.

Functional, Idiopathic, and Psychosomatic Conditions

These terms are often used when patients suffer from significant complaints without structural findings on medical examinations. Such conditions have traditionally been explained psychologically and thus marginalized in clinical practice [82]. The IFMFP model introduces an alternative understanding, where physiological disturbances in the body’s fluid and pressure systems can produce real, although microscopic and invisible, tissue changes. This provides a framework for interpreting many of these conditions as bodily responses to pressure-dynamic imbalances, not as inexplicable or “merely psychological” phenomena. Such complaints are often linked to stress and emotional strain and can be understood physiologically through the IFMFP model: prolonged sympathetic activation leads to persistent muscle tension and increased IFMFP, with resulting pain and loss of function [1,9], without being a matter of imagination. Idiopathic muscle pain, in this perspective, does not appear mysterious, but rather as a possible expression of local fluid disturbances in muscles and connective tissue. IFMFP thus provides a bodily explanation for symptoms that previously remained outside both somatic and psychological understanding. These conditions, like the others discussed here, can be explained through the IFMFP model.

Everyday Phenomena

Many adults and the elderly experience persistent muscle tension without a clear cause. This may be due to increased caution, reduced balance, or fear that pain might suddenly strike. Such unconscious protective patterns, especially in the calves, hips, back, and neck, may over time lead to sustained muscle activation and pressure buildup.

Morning stiffness is a related phenomenon. During the night, when muscles are inactive and drainage is low, fluid may accumulate and cause stiffness that improves with movement. A similar effect occurs after prolonged sitting, such as during driving or office work, where an inactive muscle pump and static tension increase tissue pressure.

Cold often triggers increased tension as an automatic protective response, which reduces drainage and may intensify pressure-related and weather-related complaints.

Taken together, such everyday reactions (unconscious activation, inactivity, morning stiffness, and cold) may trigger or reinforce pain and stiffness even without injury. The IFMFP model explains how this can happen in the absence of pathology.

Social and Identity-Related Factors

Chronic pain and fatigue conditions are influenced not only by physical mechanisms but also by social, economic, and identity-related factors. Over time, many adapt to a lifestyle and role tied to their diagnosis, characterized by a slower pace, disability benefits, and a socially accepted explanation. This can provide security yet also make recovery ambivalent: the desire for improvement may be mixed with fear of losing identity or financial stability.

The challenge often lies in the lack of realistic alternatives for transitioning from disability to active participation. Viewing disability benefits more as a form of basic income than as a reward for illness may help make recovery feel less threatening and more compatible with social belonging, joy, and meaningful activity on one’s own terms.

Myopraktikk is based on the understanding that many such conditions have complex causes, where physical overload, stress, restlessness, and life circumstances interact. Treatment, therefore, aims to identify and reduce persistent tension while helping patients realize that pain and stiffness are not necessarily signs of damage but the body’s response to overactivity. The goal is to foster balance, safety, and hope, so that recovery can mark the beginning of a more meaningful life within one’s own framework.

Interpretation of MPS in Myopraktikk

Viewed as a whole, it is plausible that a wide range of regional, systemic, pressure-related, functional, and everyday pain conditions, despite their different presentations, may share a common underlying driver in the form of increased IFMFP. It is also a likely mechanism behind many conditions that today fall between somatic and psychological understanding, where many cases of so-called “unexplained” pain may in fact be expressions of this process. When the connections between findings, clinical patterns, and physiological principles are considered, IFMFP emerges as such a clear and logical explanation that, once the pieces are put together, it is difficult to avoid recognizing the model as a central explanatory framework. This review of the clinical spectrum indicates that the IFMFP perspective can be included as a possible explanation in far more myalgias and conditions likely even more than those addressed in this article. The IFMFP model may therefore provide the basis for a substantial expansion of MPS.

Treatment in Myopraktikk

As described under the clinical spectrum, MPS encompasses a wide range of symptoms. These may present as anything from distinct, palpable tensions to generalized pain, but also, for example, as swelling, numbness, weakness, dizziness, or headache. Such manifestations, often in combination with observed asymmetries, form the clinical basis for choosing a treatment strategy. The course of the localized tension points to the relevant treatment chains and the specific treatment points that should be addressed.

Nodules, Strands, and Muscle Tension: Tactile Resolution in Real Time

Clear signs of real-time treatment response are the perceived resolution of palpable muscle knots and tensions (TrP, nodules, cords, and more general hypertonic tensions) that immediately change from palpable structures to smooth, soft tissue under the fingers. Such structures are usually painful to palpation before treatment and serve as precise reference points in mapping the patient’s tension pattern. After intervention, the same points are re-examined: if the structures are no longer palpable and the area is pain-free, this is considered a desired response. This indicates that the tissue has reacted directly and offers support for the view that these structures are not fixed tissue changes but dynamic, pressure-related fluid accumulations.

Fascial restrictions have traditionally been described as if the connective tissue itself was the primary source of limitation. Our clinical observations, however, suggest that these can disappear immediately during treatment, which is difficult to explain by structural changes in fascia. It, therefore, seems more likely that the restriction is primarily due to IFMFP-induced muscle tension.

That such accumulations appear to be released spontaneously through mechanical manipulation suggests that pressure relief in the body’s intrafasciomembranal fluid compartments is central. This also provides a rare window into the real-time effect of biotensegrity. It may be postulated that neurogenic activation in this context appears as a contributing response rather than a primary driver.

The crosswise traction is applied to elicit local, systemic, and remote responses. The effect appears to follow treatment chains that carry a baseline tonus, enabling the spread of vibration and resonance (tissue communication) throughout the structure. This may influence tension and pressure in peripheral areas through biomechanical, biochemical, and bioelectrical mechanisms.

It is precisely this holistic influence through functional treatment chains that distinguishes Myopraktikk from therapies with a purely local focus.

A Holistic Intervention: From Local Manipulation to Systemic Response

When a treatment point within a chain is manipulated (most often on muscles located at the scapula), this often triggers immediate changes in distant areas. For example, the lumbar part of the erector spinae may feel softer after treatment, with reduced tonus and loss of palpable structures. Similarly, both patient and practitioner may notice less tension and hypertonicity in the gastrocnemius of the calf. Such findings are interpreted as signs of redistribution of pressure and tension throughout the body’s network. Previous studies have described similar remote responses [83,84], supporting the theory of regulation through the fascial and biotensegral network, where pressure, fluid dynamics, and structural tension interact through multiple simultaneous mechanisms.

Restoration of Permeability

Manual mechanical treatment, such as friction, manipulation, or oscillation-like stimuli, is thought to reorganize the structure and break up HA aggregates [85]. Myopraktikk manipulation likewise induces such oscillations or vibrations in the tense fasciomembrane, directly affecting HA. The vibrations relieve the shutting effect within the membrane and contribute to the reorganization of HA, both within the membrane and in the HA layer on the inside, between fascia and underlying muscle. This phenomenon can be compared to shaking a sieve: without movement, no passage occurs, but when shaken, the particles pass through. In the same way, vibrations during treatment can dissolve structural resistance, increase permeability through the fasciomembrane, and restore fluid flow [12].

Pressure Relief and Drainage

This initial, immediate pressure reduction across the fasciomembrane further alleviates mechanical compression of vessels and nerves, lowering resistance and easing obstruction to both venous and lymphatic drainage. As a result, fluid can once again circulate freely, and the previous overpressure is redistributed and normalized. This process explains why an immediate reduction in palpable structures and tensions can be observed during treatment.

Remote Response via Biotensegrity, Fluid Flow, and Micro-signals

A key element in this understanding is that mechanical manipulation, particularly when sufficient, transverse, and targeted, can generate vibrations and micro-movements that propagate through the body’s fascial connections, exactly because the tissue is part of a pre-tensioned network that allows such vibrations to travel across regions. Manipulation within a treatment chain may therefore create a form of fascial resonance, where mechanical vibrations along the chain restore fascial membrane permeability and normalize IFMFP, even at distant sites.

Tension and fluid pressure are likewise not isolated phenomena. They are continuously distributed throughout the body’s interconnected structures, where force and pressure shift and redistribute in response to local changes. Through biotensegrity’s self-stabilizing load distribution, balance is restored [35]. Releasing a single point can therefore trigger adjustments elsewhere, such as changes in posture, movement, or even the perception of pain in areas that were not directly treated. The body, in this sense, can be likened to a waterbed with multiple interconnected chambers, where local pressure changes spread rapidly, both mechanically and through fluid dynamics.

Increased or Decreased Tonus: Reduced Asymmetries

As previously described, the self-regulating system of biotensegrity continuously seeks to maintain even tension and load distribution [35]. When a blockage is released, tonus may be redistributed; some areas decrease, while others increase. For example, low tonus in one calf may rise when the opposite side is treated, which, in clinical observations, appears exactly as the body’s attempt to restore balance. Increased tonus after treatment should therefore be understood as systemic regulation, not as a dysfunction.

When IFMFP increases in one muscle, mechanical tension is exerted on neighboring tissues and associated structures [9]. Conversely, when the pressure is reduced, these same connections can be released, allowing tension to redistribute and the system to reorganize. Fluid dynamics may also contribute to changes in pressure and tonus within adjacent tissues. In addition, proprioception, reflex mechanisms, and responses from the autonomic nervous system can help adjust tension as a protective reaction [86-88]. Clinically, this regulation and self-stabilization are reflected in a reduction of asymmetries. When tensions are released and the body regains its natural tension pattern, the entire process can be understood as a form of reset, an expression of the system’s capacity for self-regulation and stabilization [35].

Field Communication and Micro-signals in Remote Response

As mentioned, the Myopraktikk treatment chains coordinate movement, force, and tension. For this coordination to function, the body must simultaneously restrict how forces and signals spread. Without such limitations, mechanical, chemical, and electrical impulses would diffuse freely, and controlled interaction would be lost. It is therefore logical to assume that electrokinetic and fluid-dynamic signals also follow these same treatment chains.

A growing understanding within cell biology and biophysics suggests that mechanical deformation of cells, such as during Myopraktikk treatment, can generate microscopic electrical and electromagnetic fields [8,89].

When crosswise traction is applied to a fascial area, it produces not only local deformation of cells and tissues, but also mechanical waves that can propagate through the fluid-filled myofascial tissue [85]. Such waves generate micro-movements in both collagen fibers and surrounding cells. Deformation of cells and fibers thus occurs not only in the area directly manipulated, but also in tissue reached by these mechanical pressure waves. This deformation activates mechanosensitive openings in the cell membrane (ion channels known as Piezo1 and Piezo2). These channels open when cells are deformed by stretching or pushing, allowing calcium ions (Ca²⁺) to enter. This creates an electrical current and alters the electrical potential difference between the inside and outside of the cell (the membrane potential). Such changes can trigger intracellular signaling pathways or propagate to neighboring cells [89,90]. At the nanoscale, collagen fibers have also been shown to possess piezoelectric properties, meaning that mechanical deformation can generate changes in electrical charge in these structures as well [91]. The cytoskeleton consists of filamentous proteins (actin, microtubules, and intermediate filaments) that provide the cell with shape and strength. When these proteins are stretched or bent, they transmit physical forces to different parts of the cell, and because the proteins and surrounding membranes are charged, the deformation can alter the distribution of charges and give rise to yet another type of small electrical currents [89]. In addition, the water-rich fasciomembranes surrounding the muscle fibers are also deformed during manipulation. This forces ions and fluid through the charged ECM and can create yet another source of bioelectricity, known as “streaming potentials” [92].

When a cell is exposed to such mechanical deformation from manipulation or waves of pressure, a local electromagnetic field may be generated that propagates to nearby cells. The receiving cells respond with their own deformation and emit a new field, creating a cascade of micro-signals and micro-movements that spread rapidly and in a coordinated manner through the tissue [89]. In addition, such deformation also generates small electrical signals or chemical signaling molecules within the cell. These can be transmitted directly to neighboring cells through small openings in the cell membrane, allowing the signal to jump from cell to cell without first leaking into the surrounding tissue [89]. This thus provides two different communication pathways between cells in the tissue: electromagnetic fields and direct cell-to-cell transmission via signaling molecules.

A parallel mechanism is that protein channels in the cell membranes open when deformed during treatment, causing ATP to leak out and trigger Ca²⁺ waves between neighboring cells through adenosine triphosphate (ATP)-based cell signaling [93]. This also creates a field of activation between cells in the tissue and may contribute to synchronous response patterns within the treatment chain.

In this way, a Myopraktikk manipulation can rapidly be translated into fields, providing a potential basis for systemic and integrated mechanical, chemical, and electrical response patterns, where many cells may react almost simultaneously, much like when one bird changes direction and the entire flock follows immediately. Such signals can propagate through interstitial fluids, fasciomembranes, muscle tissue, and vessels, as well as be transmitted to and from the nervous system, which is sensitive to changes in electrical fields.

Accordingly, the direction of such signals is likely governed by the body’s anatomy and organization, reaching all the way to distant regions within the Myopraktikk treatment chain. When the signals arrive at an area with elevated IFMFP, muscle cells, fascial cells, and fibers may respond with small changes in tension, creating micro-vibrations and increased mobility within the fasciomembrane while at the same time reorganizing the HA in and the layer inside the fasciomembranes (a systemic “shaking of the sieve”). Here as well, the permeability of the fasciomembrane increases, reducing the initial peak pressure and thereby lowering obstruction of venous and lymphatic drainage of IFMFP.

Such microscopic oscillations or vibrations may also occur when adrenaline and noradrenaline are released during treatment [94], imperceptible, yet with potentially significant mechanical effects. Adrenaline-driven micro-tremors may, therefore, likewise increase the permeability of fasciomembranes and relieve IFMFP in a similar way.

Field communication and micro-signals, together with biotensegrity and fluid dynamics, may thus explain what is clinically observed as tactile remote responses, and further clarify how treatment at one site can influence tissue at another, without direct anatomical contact. Clinical experience suggests that one precise and deep manipulation often has a greater effect than many mild interventions. Conversely, experience also indicates that treatment that is too weak often produces little or no real effect. Precise and sufficient manipulations, on the other hand, can overcome this resistance and trigger an immediate reorganization of tissue, pressure, and tension throughout the Myopraktikk treatment chain.

When all this is placed into a therapeutic context, it becomes clear that both the degree and the type of manipulation may greatly influence how far and how deeply the response spreads through the body. Deeper mechanical manipulation generates more energy, creating more powerful and far-reaching pressure waves in the tissue, which can reach more cells and fibers, thereby also producing more bioelectric activity that, in turn, affects more distant regions of the treatment chains. This may further trigger neural activity, reflex responses, or autonomic reactions, thereby serving as a bridge between mechanical stimulation and systemic regulation.

Speculative Ideas

Several mechanisms remain largely unexplored, from alternative transport pathways to bioelectrical interactions. Future research may even consider whether quantum biological phenomena could contribute to systemic coordination [7]. This suggests that we may have only begun to touch the complexity of bodily communication, which may prove far more significant than is currently understood. Some researchers have suggested that fascia may play a role in the transport of biophotons and, due to its high degree of collagen organization, may exhibit properties similar to fiber optics [95,96]. This may support the notion that electromagnetic signals can potentially reach distant cells and influence tissues that are not directly connected anatomically, entirely independent of the nervous system.

Autonomic reactions and bodily regulation

During such deep Myopraktikk treatment, reactions such as trembling, laughter, crying, or cold sweating may also occur [8], suddenly and without being initiated by the patient. These reactions are relatively uncommon, but when they appear, treatment is paused to allow the patient to recover and regain composure. Patients are informed in advance that such reactions may occur, which generally contributes to a sense of safety and reassurance. These reactions are usually benign and resolve; however, if a patient becomes distressed or the reaction appears atypical, treatment should not be continued, and referral considered. Such reactions do not appear to be directly related to the degree of local muscle tension, but rather point to overarching regulatory mechanisms in which the body responds as a whole.

When such autonomic reactions occur, pressure and touch are first registered by mechanoreceptors in the skin, muscles, and fascia, and are then interpreted in the brain as signals concerning the entire body [97,98]. Thus, physical treatment can activate both sympathetic and parasympathetic responses, often with the vagus nerve as a central mediator of parasympathetic regulation [99]. Trembling can be understood as a transitional reaction, where the muscles shake off tension as the body shifts from activation to relaxation [98]. Crying often begins with sympathetic mobilization, involving faster breathing and emotional release, but is followed by parasympathetic calm that brings relief and clarity [100]. Laughter can similarly be triggered by a brief sympathetic “spark” but ends with parasympathetic recovery, which explains why the patient often feels calmer afterward [101]. Cold sweating is more directly linked to sympathetic activation, through neural pathways controlling the sweat glands [102], but may also form part of a broader regulatory cycle in which parasympathetic activity subsequently takes over. Such “fight, flight, freeze” responses can therefore be interpreted as the body’s way of releasing accumulated tension and pressure, a spontaneous physical and emotional discharge [98]. Patients without obvious autonomic reactions can also report a sense of relief or emotional clarity after treatment, suggesting that such regulation may occur without visible expression. This can be understood as a form of systemic reset and as a natural expression of the body’s regulatory mechanisms, not as pathology.

The clinical dialogue: Preparing the patient

A central part of the overall treatment strategy is what happens before the manual intervention itself. The patient receives thorough information about the treatment, as well as an understandable introduction to how the body functions as a system, with fluid pressure, tensions, and regulatory mechanisms, and how both physical and psychological stressors can lead to persistent muscle tension and pain. This creates a sense of safety, reduces catastrophic thinking, and provides an explanation the patient can relate to, often fostering both trust and hope.

When the patient understands how and why their symptoms arise, it becomes easier to identify and avoid activities, situations, and thought patterns that previously contributed to sustained activation and increased IFMFP. While the treatment helps release tension, it is often the insight and understanding that prevent these patterns from recurring. When underlying causes are clarified, and simple strategies for coping and meaningful future perspectives are developed together with the patient, regulation is more easily maintained over time, and the risk of relapse is reduced.

In contrast to many traditional approaches, which often emphasize discipline and lifestyle change, Myopraktikk emphasizes safety, mastery, and enjoyable activities that almost automatically support relaxed musculature. Many patients experience failure when faced with admonitions and demands, something that in itself reinforces underlying stress. By focusing instead on what brings joy and genuinely works in the patient’s everyday life, a new space for mastery and control is created, grounded in trust in the patient’s own body rather than in a sense of inadequacy.

The patient’s role in relapse prevention

To prevent relapse, the patient is given recommendations on how the body can be regulated and supported in daily life. These include both physical and psychological measures.

Important advice for physical overload is calm, repetitive movements, the so-called “cooldown” training, after exertion. An enjoyable walk, light cycling, or other low-intensity activity with minimal muscle tension activates the muscle-venous/lymphatic pump, promotes fluid drainage, and normalizes pressure [103]. An example can be seen in athletes: after overtraining or intense exertion with strong muscle contractions that pump the muscles and inhibit fluid drainage, IFMFP remains high. Cooldown (repetitive, low-tension movements), on the other hand, can facilitate drainage.

For many patients, especially those with stress-related or psychosomatic complaints, understanding their own tension patterns is a crucial part of treatment. It involves recognizing the connections between life situation, relationships, expectations, and the body that carries it all. In dialogue with the myopraktor, specific events, patterns, or thoughts are often identified as “stored in the body.” When patients understand what is happening and how it affects them, they can make small changes that have a major effect: more calm, less struggle, more space for breathing and presence, without adding guilt.

In line with this, patients are encouraged to seek pleasant and safe social or physical activities, whatever brings comfort and well-being, not necessarily stillness, but experiences where the body can do something good. When the body feels safe and engaged in something positive, it almost automatically releases tension, such as “raised shoulders,” often without the patient even noticing. The recommendation is therefore not to do less, but to do differently: to move gently, without performance pressure or old patterns of physical or psychological strain.

Discussion

The clinical observations underlying Myopraktikk point toward a new understanding of muscle pain and functional disorders. Particularly striking is that palpable structures such as “knots,” “cords,” and hypertonic tensions often disappear instantly during treatment and that this effect also occurs in remote areas. This challenges traditional models based on local tissue injury and supports a more systemic and functional explanatory framework.

The IFMFP model emerges in this context as a central unifying mechanism. By understanding muscular tension states as expressions of fluid-dynamic imbalance, rather than structural damage, a framework is created that explains both local and systemic symptoms, even in the absence of pathological findings on imaging. This perspective provides a new understanding of conditions such as TrP, DOMS, OTS, CECS, and MPS, and opens the possibility that several seemingly different disorders, such as fibromyalgia, ME/CFS, PTSD, and IBS, may represent variations of the same underlying physiological process. MPS may, in this sense, be considered an overarching umbrella diagnosis for pressure-related muscle- and fascia-associated pain, with IFMFP as the common denominator.

A distinctive feature of the IFMFP model is that it integrates a number of well-established physiological principles into a single coherent explanation: that arterial supply has higher pressure than venous drainage, that sustained muscle activation can produce venous stasis and fluid accumulation, and that low pH influences hyaluronan and its properties, with experimental studies showing increased viscosity [21]. These are not new or speculative phenomena, but well-known mechanisms within tissue physiology, here combined in a new context. Clinically, this may be inferred when fascia feels stiff and loses gliding, and reduced permeability of fasciomembranes can amplify pressure problems.

The model also offers plausible explanations for several otherwise poorly understood phenomena, such as weather-related pain, immediate structural changes during treatment, and systemic reactions such as remote responses. Such reactions can be understood as expressions of the body’s ability to reorganize itself in real time through mechanical, electrical, and biochemical signaling pathways. Here, the principles of biotensegrity provide a structural-mechanical explanation: the body functions as an integrated network in which a change in one part automatically affects the rest. At the same time, recent research points to field communication as a possible explanation for rapid signal transmission and coordination within tissue, where electrical and electromagnetic impulses are conveyed through the ECM and fascial structures.

An important contribution of the IFMFP model is that it provides a physiological explanation of what terms such as muscle knot, TrP, and muscle tension actually represent: a fluid-based pressure increase and altered tissue properties in various fasciomembranal compartments. This creates a new understanding of what is physically “stored in the body” through load and stress, and puts words to a bodily phenomenon that until now has been largely unclear both clinically and theoretically.

Furthermore, the model raises the question of where the line should be drawn between normal adaptation and emerging dysfunction. Increased muscle mass and connective tissue thickening are in themselves normal adaptations to load. However, when pressure, low pH, and impaired drainage disrupt the body’s ability to self-regulate, this may develop into persistent states of tension with loss of function, even in the absence of visible damage.

Clinical observations also suggest that the precision and intensity of treatment play a crucial role in triggering the desired response. Targeted and sufficiently mechanical stimulations appear capable of influencing tissue permeability and pressure balance, thereby activating the body’s regulatory mechanisms. This supports the view that remote responses and systemic changes are not solely due to local effects but are triggered as part of a holistic reorganization.

Limitations

This article is presented as a narrative review and conceptual hypothesis. The clinical observations described are based on palpation, visual assessment, and patients’ subjective feedback. While these reports are commonly reported by patients and clinically valuable, they remain subjective and are not supported by quantitative measures such as the visual analog scale (VAS), range of motion (ROM), or ultrasound. No blinding or control groups were applied. These limitations highlight the need for systematic and objective studies to further test and validate the concepts discussed.

## Conclusions

This article has presented Myopraktikk (NO) within a narrative review and conceptual hypothesis on IFMFP, biotensegrity, and immediate remote myofascial responses. The framework is grounded in clinical observations: palpatory assessments, visual inspection, and patient feedback. By reframing muscular and fascial disorders as dynamic manifestations of pressure, fluid, and tension within interconnected compartments, the model provides a framework for understanding a wide spectrum of pain and stiffness conditions. These range from local trigger points to complex systemic syndromes such as fibromyalgia, OTS, ME/CFS, PTSD, IBS, and neuropathies, where pressure- and tension-related mechanisms may contribute to the symptom picture. It also provides plausible accounts for everyday phenomena such as morning stiffness, weather-related pain, and stress-induced body tension, suggesting that MPS may represent an overarching umbrella diagnosis for pressure- and tension-related mechanisms.

Observations further suggest that palpable structures and tensions can vanish instantly, not only at the treatment site but also at distant regions, suggesting immediate remote responses that challenge conventional explanations of musculoskeletal disorders. Autonomic treatment reactions and systemic “reset” phenomena further highlight the body’s capacity for rapid, whole-body reorganization once pressure barriers are relieved. The body thereby appears as a highly organized, multidimensional information field, where local intervention can produce global effects, and treatment must engage with both wholeness and complexity. Within this framework, Myopraktikk emerges as a comprehensive paradigm for understanding and treating myalgias, calling for further research to document remote responses, quantify IFMFP, and explore pressure propagation through fascia, treatment chains, and biotensegrity networks.
